# Fluid management for dengue in children

**DOI:** 10.1179/2046904712Z.00000000051

**Published:** 2012-05

**Authors:** Nguyen Thanh Hung

**Affiliations:** Department of Dengue Hemorrhagic Fever, Children’s Hospital No. 1, Ho Chi Minh City, Viet Nam

**Keywords:** Dengue, Dengue haemorrhagic fever, Fluid, Management, Clinical

## Abstract

Dengue is a serious public health problem worldwide. Dengue shock syndrome (DSS), the severe form of dengue fever, can cause death within 12–24 hours if appropriate treatment is not promptly administered. For patients with DSS and the 30% of non-shocked dengue patients who require intravenous fluid therapy, a range of solutions is available for plasma volume support. Crystalloid solutions, such as normal 0·9% saline or Ringer’s lactate, are the ones most commonly used. In severe cases, colloid solutions may be administered for their greater osmotic effect, although they carry a greater risk of adverse events. This paper summarises the key clinical data, comparing fluid regimens in children with severe dengue, and concludes that the majority of patients with DSS can be treated successfully with isotonic crystalloid solutions. If a colloid is thought necessary, a medium-molecular-weight preparation that combines good initial plasma volume support with good intravascular persistence and an acceptable side-effect profile is optimal. Further research should aim to determine whether there are benefits to early treatment with colloids, and which colloid solution is most effective for resuscitation of DSS patients.

## Principles of Fluid Management for Dengue in Children

Dengue infection causes a broad spectrum of clinical disease, which can range in severity from febrile illness to serious bleeding and shock. Two major pathophysiological responses are associated with severe dengue infection – plasma leakage leading to hypovolaemic shock and/or abnormal haemostasis leading to haemorrhage.1,^2^

**Figure 1:**
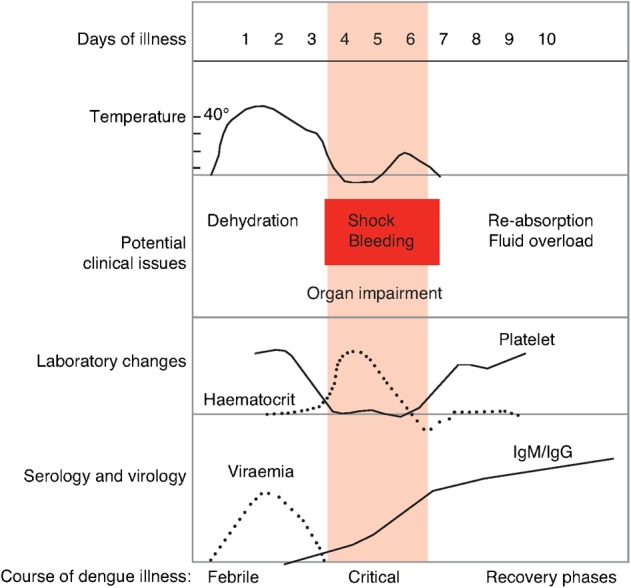
Clinical course of dengue^1^

The clinical course of dengue includes febrile, critical and recovery phases (Fig. 1), and there are different challenges for fluid management at each stage.^1^ In the initial febrile stage, the aim is to treat dehydration. The majority (70%) of non-shocked dengue patients can be treated as outpatients with oral rehydration regimens; however, the remaining 30% of these patients and all DSS patients require intravenous (IV) fluid therapy.^3^

During the critical stage, there is an increase in capillary permeability and shock can result if a large volume of plasma is lost through leakage. The recommended regimen for the treatment of DSS is: immediate and rapid replacement of the plasma loss with isotonic crystalloid solutions or, in the case of profound shock, colloid solutions; continued replacement of further plasma losses to maintain effective circulation for 24–48 hours; correction of metabolic and electrolyte disturbances; and blood transfusion in cases with severe bleeding. If large amounts of fluid are required, these should be reduced gradually as plasma leakage decreases in order to prevent hypervolaemia, an excess in plasma volume which can cause oedema, respiratory distress or congestive heart failure, during the recovery stage.1,^2^

## Choice of Intravenous Fluid

Replacement of plasma lost because of increased vascular permeability is a mainstay of severe dengue management, particularly during the critical stage.^3^ Two main types of volume expander are used to replace lost fluid in the management of dengue fever: crystalloids and colloids.^1^ Crystalloids are aqueous solutions of mineral salts or other water-soluble molecules, whereas colloids contain larger insoluble molecules such as gelatin, dextrans or starches.

The most commonly used crystalloid is 0·9%, or ‘normal’ saline, a hypertonic solution with an osmolality of 308 mOsm/L that has higher sodium and chloride levels than normal plasma. Normal saline is a suitable option for initial fluid resuscitation but repeated large volumes of 0·9% saline may lead to hyperchloraemic acidosis and decreased blood pH owing to excessive chloride levels. Therefore, if serum chloride begins to exceed the normal range, other alternatives such as Ringer’s lactate may be preferable.^1^

Ringer’s lactate has lower sodium and chloride contents than 0·9% saline, and an osmolality of 273 mOsm/L. It should be avoided, however, in individuals with liver failure as they have a reduced ability to metabolise lactate.

The most common types of colloid used for plasma volume support are gelatin-, dextran- and starch-based solutions (summarised in Table 1).^4^ In contrast with crystalloid solutions, colloid infusion can expand volume in excess of the actual volume administered and so may be beneficial for rapid fluid delivery for emergency resuscitation of hypovolaemic shock. Furthermore, colloid molecules may show increased efficacy since they increase plasma oncotic pressure, thereby altering the flux of fluid across the capillary membrane and drawing fluid back into the capillary from the interstitial space.

**Table 1:** Characteristics of colloids used for plasma volume support^4^

	Initial volume expansion (%)*	Duration of volume effect (hrs)	Adverse effect on coagulation	Allergic potential	Other significant side-effects
3% Gelatine (MW = 35,000)	60–80	3–4	+/−	++	
10% Dextran 40 (MW = 40,000)	170–180	4–6	++	+	Renal failure in hypovolaemic patients
6% Dextran 70 (MW = 70,000)	100–140	6–8	++	+	
6% Hydroxyethyl starch (MW = 200,000/0·5)	100–140	6–8	+	+/−	
6% Hydroxyethyl starch (MW = 400,000)	80–100	12–24	++	+	

Management of dengue; Wills B. Halstead SB (Ed.) Copyright 2008 Imperial College Press

*Infused volume

MW, molecular weight

One of the greatest concerns regarding colloid use is the impact on coagulation. Dextrans theoretically bind to von Willebrand factor/Factor VIII complex and impair coagulation; however, this has not been observed to be of clinical significance in fluid resuscitation of dengue shock patients.^1^ Gelatin has a lesser effect on coagulation but the highest risk of allergic reaction. Allergic reactions have also been observed in patients treated with dextran 70 and dextran 40, and can potentially cause osmotic renal injury in hypovolaemic patients.6,^7^

Care should be exercised in storing colloid solutions, particularly in warmer climates, since dextrans and gelatins are very sensitive to temperatures exceeding 20–30°C which can cause degradation into smaller molecules.^8^ In clinical terms, this would decrease the solutions’ intravascular volume effect and increase their renal elimination.

## Randomised Controlled Trials of Fluid Management in Dengue Fever

A number of randomised controlled trials have been undertaken to compare the efficacy of different fluid regimens in managing DSS in children. A pilot study involving 50 children with DSS showed minor differences in the immediate clinical responses to different fluids.^9^ Children were randomised to receive either normal saline (*n* = 12), Ringer’s lactate (*n* = 13), dextran 70 (*n* = 12) or 3% gelatin (*n* = 13). In a pooled comparison of crystalloids and colloids, patients who had received colloid infusions had significantly greater increases in mean haematocrit (*P* = 0·01), blood pressure (*P* = 0·005), pulse pressure (*P* = 0·02) and cardiac index (*P* = 0·02). In individual comparisons, dextran 70 was found to be the most effective solution for improving cardiac index and haematocrit.

A larger study of 230 children with DSS that compared the same four fluids initially suggested improved pulse pressure recovery time following early treatment with colloids.^10^ However, pulse pressure at presentation was identified as a potential confounder, and, when only the most severe cases (presenting pulse pressure ⩽10 mmHg, *n* = 51) were compared, fewer differences were found. Of children who received gelatin, significantly fewer had a recovery time of more than 1 hour compared to those who received Ringer’s lactate (*P* = 0·017). Comparisons between all other solutions were not significant. This study suggests that the majority of patients with DSS have mild-to-moderate shock and will respond well to conventional treatment with crystalloids. A small minority with more serious disease may require more aggressive management with colloids from the outset. However, this study was statistically underpowered and the differences in presenting pulse pressure may have obscured some of the results; for example, any benefit associated with 3% gelatin would be expected to be more pronounced with dextran 70, but there were very few severe patients to compare in the dextran 70 group. Further large-scale studies, stratified for admission pulse pressure, were recommended.

The largest randomised study to date included two arms, with patients stratified for presenting pulse pressure.^11^ Children with moderately severe shock (pulse pressure >10 to ⩽20 mmHg, *n* = 383) were randomised to receive Ringer’s lactate (*n* = 128), 6% dextran 70 (*n* = 126) or 6% hydroxyethyl starch (HES) 200/0·5 (*n* = 129). Ringer’s lactate was found to be as effective as colloid therapy on the primary outcome measure of requirement for colloid rescue or fluid resuscitation. However, patients treated with Ringer’s lactate took longer to achieve cardiovascular stability than patients receiving either colloid (*P* = 0·02).

A further 129 children with severe shock (pulse pressure ⩽10 mmHg) were randomised to receive one of the colloids – dextran 70 (*n* = 67) or HES (*n* = 62) – and both colloid preparations performed equally well with regard to cardiovascular stability and the number of patients who required resuscitation. However, the dextran preparation was associated with more adverse events than HES, with 8·0 *vs* 0·5%, respectively, reporting allergic-type reactions including transient high fever and rigors. These adverse events responded to symptomatic treatment alone and there were no differences in severe adverse events such as significant new bleeding or clinical fluid overload.

This study indicated that Ringer’s lactate, the cheapest and safest preparation available, was the best treatment for moderate shock in children with DSS and early intervention with colloids was not necessary. A clinical trial that compared normal saline and lactate solutions in a large, heterogeneous population in intensive care suggested that these fluids are equally effective.^12^ For children with severe shock, there were no clear advantages of dextran over starch solutions, but starch may be preferred for its lower adverse event profile.

A study of 104 DHF patients with severe plasma leakage who had failed to respond to crystalloids and required fluid resuscitation compared bolus doses of two colloids, 10% dextran 40 (*n* = 57) and 10% HAES-steril (*n* = 47), for their effectiveness, impact on renal function and haemostasis and any complications.^13^ HAES-steril was found to be as effective as dextran 40 on measures of haematocrit change and in terms of the number of doses and volume of fluid required. Both colloidal solutions were deemed safe in these patients; there were no allergic reactions or interference with renal function or haemostasis.

## Conclusions

Taken together, these studies show that the majority of children with DSS can be treated successfully with isotonic crystalloid solutions. If a colloid is considered necessary, clinicians must continue to rely on personal experience, familiarity with particular products, local availability and cost. A medium-molecular-weight preparation that combines good initial plasma volume support with good intravascular persistence and an acceptable tolerability profile is probably the optimal choice.

It should be noted that the positive overall outcomes described in these studies probably reflect the quality of care as much as the interventions themselves. Hourly observations and immediate access to haematocrit testing coupled with a conservative intervention policy allow fluid requirements to be met as early as possible and carefully titrated. In more poorly resourced settings, intensive care provision is more challenging and complications such as fluid overload are more likely to occur.

Further research using higher patient numbers, stratified for severity, will be needed to determine whether early treatment with a colloid confers a true advantage in those with severe shock, and which colloid solution is most effective for resuscitation of DSS patients.
